# Cosmic time synchronizer (CTS) for wireless and precise time synchronization using extended air showers

**DOI:** 10.1038/s41598-022-11104-z

**Published:** 2022-04-30

**Authors:** Hiroyuki K. M. Tanaka

**Affiliations:** University of Tokyo, Tokyo, Japan

**Keywords:** Experimental particle physics, Physical oceanography, Seismology, Volcanology, Civil engineering, Electrical and electronic engineering

## Abstract

Precise time synchronization is an essential technique required for financial transaction systems, industrial automation and control systems, as well as land and ocean observation networks. However, the time synchronization signals based on the global-positioning-system (GPS), or global-navigation-satellite-system, are sometimes unavailable or only partially available in indoor, underground and underwater environments. In this work, the simultaneous and penetrative natures of the muon component of the extended air shower (EAS) were used as signals for time synchronization in environments with little or no GPS coverage. CTS was modeled by combining the results of previous EAS experiments with OCXO holdover precision measurements. The results have shown the capability of CTS to reach perpetual local time synchronization levels of less than 100 ns with a hypothetical detector areal coverage of larger than 2 × 10^−4^. We anticipate this level of areal coverage is attainable and cost-effective for use in consumer smartphone networks and dense underwater sensor networks.

## Introduction

Fifth-generation (5G) mobile/cellular radio access network (RAN)^1^ systems, industrial automation and control systems^2^, as well as land^3^ and ocean^4^ observation networks all require real-time connectivity with precise time synchronization in order to provide robust reference time information to the devises located in these networks on a common time basis with a jitter level below 1 microsecond^1^. Such requirements are typically fulfilled through wired technologies like Time-sensitive Networking (TSN)^5^. TSN provides guaranteed IEEE-802.1-based real-time data delivery with precise time synchronization. Moreover, recent advancements have been made in fiber optic time and frequency techniques that allow almost perfect compensation of time delay or phase fluctuations when operated bidirectionally over the same optical fibers to enable time synchronization with precision ranging from 10 ps to below 1 ns depending on the link length and the technology used^6–10^. While wireless technologies offer various benefits for network communication^11,12^, accuracy is one of the most important concerns. For example, since seismological and volcanological observations with a seismometer array require the seismic wave sampling rate to be more than 1 kHz, in this case, a wireless time synchronization accuracy of less than 10 microseconds would be required^3^. Wireless devices can achieve perfect time alignment to coordinated universal time (UTC) by using global positioning system (GPS)/global navigation satellite system (GNSS) receivers. Currently, a 2-ns precision level is achievable with the GPS-based time transfer links^13^ and even a 1-ns precision level can be achieved with a new state-of-the-art method for calibrating receivers^14^. Furthermore, two-way satellite time and frequency transfer (TWSTFT) links with geo-stationary satellites could improve this accuracy up to sub-nanosecond levels^15^. However, this solution does not work when GPS signals are not available or when GPS signals are only partially available (e.g., polar, indoor, mountainous areas, underground or underwater environments) or when the GPS network nodes malfunction (e.g., receiving signals from different GPS satellites or temporal shift of GPS satellites). Moreover, if we equip GPS receivers to all of the network nodes, the total power consumption increases and as a consequence, the battery drains faster. Reliable battery performance, particularly maintaining longer durations of performance between battery charging sessions, is a critical issue for field measurements in particular.

The requirements for effective wireless synchronicity for industrial use have been summarized by several researchers^16,17^. Possible approaches have been categorized into three classes. Class (I): remote control and monitoring, Class (II): mobile robotics and process control, and Class (III): closed loop motion control. For Classes (I), (II), and (III), synchronicity with accuracies less than 1 s, 1 ms, 1 µs, are respectively required. In order to respond to these requirements, there have been various WLAN-based research approaches addressing wireless time synchronization techniques including the reference broadcast infrastructure synchronization protocol method, which realized an accuracy of 200 ns–3 µs^18^, the adaptive synchronization in multi-hop time-slotted-channel-hopping (TSCH) networks method, which realized an accuracy of 76 µs^19^, the temperature-assisted clock synchronization method, which realized an accuracy of 15 µs^20^, and a time synchronization method based on the second order linear consensus algorithm, which realized an accuracy of 1 µs^21^. Other techniques include the dynamic stochastic time synchronization method, which realized an accuracy of approximately 8 µs with a Kalman filter (KF) estimator^22^, and the fine-grained network time synchronization 6.29 µs with a linear regression (LR) estimator^23^. Pros and cons exist for all these techniques. Since all of these aforementioned techniques utilize electromagnetic waves for communications, relatively small sized devices can be facilitated. However, in order to avoid communication failure due to noises and collisions, generally the automatic repeat-request (ARQ) mechanism and communication latency should be included in these techniques; hence degrading the synchronization quality. On the other hand, since the currently proposed technique utilizes naturally occurring multiple particles that arrive throughout the globe at the same time, such communication failure and message collisions do not take place. However, a larger device size, in comparison to those used in WLAN techniques, would probably be required due to the limited flux of cosmic rays.

In underwater environments, the situation is harsher since WLAN techniques cannot be used in water. If radio-based networks for general computing or sensor networks are compared with short-range acoustic networks, one can find that propagation delay is much larger due to a large difference between the speed of light (a few hundred thousand km/s) and the speed of sound in water (1500 m/s)^24^. Recently, a wireless sensor network for high time-resolution (ns-scale) has been designed, consisting of sensor nodes which are synchronized to within 1 ns by using periodic, high intensity optical pulses from light-emitting-diode (LED) bursts^25^. However, in this scheme, empty space is required between nodes, and thus it is difficult to practically use this technique in an environment such as inside commercial buildings, underwater, or in an underground complex. One possibility to solve this problem is to use an atomic clock to provide backup timing signals when the GPS signal is lost. For example, a commercially available cesium oscillator provides stable timing information with a drift level of only 100 ns in 14 days. However, the extremely high cost of the atomic clock (over 300 k USD) hardware restricts its wide-scale use^26^. Another possibility to temporary solve this problem is "holdover"^27^. Synchronization standards have defined the term “holdover” to refer to when the network continues to function reliably even when the synchronization input (e.g., GPS/GNSS signals) has been disrupted or becomes temporarily unavailable. For this purpose, the oven-controlled crystal oscillator (OCXO) has been industrialized to provide reliable and accurate holdover measurement capability; this can be utilized during moments the GPS/GNSS receiver doesn’t pick up a signal. However, the drift level of the OCXO is much higher than the atomic clock and is typically limited to 0.5 microseconds per hour^27^, meaning the timing may deviate by more than 1 microsecond in 24 h. If a non-GPS synchronization input could be provided to OCXO frequently, then the devices in the network could be synchronized more precisely and consistently.

The muonic component of an extended air shower (EAS) has been used to estimate the energy and mass of its primary cosmic rays^28,29^. An EAS can be measured by sampling multiple showers of secondary particles at ground level with 2-dimensionally dispersed detector arrays such as KASCADE^30^, GREX/COVER_PLASTEX^31^, and AKENO^32^. Since primary cosmic rays arrive at a velocity near the speed of light, the resultant secondary particles generated in the atmosphere tend to move in generally the same direction as the primaries, and arrive at the ground level at almost the same time (this time structure is labeled hereafter as EAS time structure); however, the shower particles spread slightly sidewise as they travel towards the ground surface, generating a specific and recognizable spatial extent of shower particles at the ground level (this spatial extent will be labeled hereafter as the EAS disk). Muons, one of the shower particles, are in general produced near the tropopause; however, they scatter far less than electromagnetic (EM) particles do and thus, their paths to the Earth’s surface are usually straight. On the contrary, EM particles reach ground level after undergoing multiple scattering processes. As a consequence, their path lengths are longer than muon's path lengths and thus, each has a longer time of flight (TOF). As a result, the muon components arrive earlier at ground level than the EM components. The time structure of the EAS disk has been extensively studied for muons with energies more than 10 PeV, and the averaged arrival time and the disk thickness (standard deviation of the particle arrival time distribution) have been measured as a function of the distance from the shower axis, and it was found that inside an EAS disk area measuring less than 200 m from the shower axis these muons arrive within the time range of 50 ns^33^.

Cosmic-ray muons are highly penetrative particles, and muography takes advantage of the characteristics of muons, particularly their penetrative nature and universality, for a wide variety of applications, including visualizing the internal structure of volcanoes^34,35^, ocean^36^, railway tunnels^37^, natural caves^38^, and cultural heritage^39^ worldwide. Likewise, by utilizing its universality and relativistic nature, cosmic-ray muons can be used for underwater or underground navigations^40^. This paper proposes a novel wireless time synchronization technique which takes advantage of the characteristics of EAS particles, the predictable nature of their arrival to the Earth’s surface (in conjunction with the OCXO), to provide stable and accurate time synchronization without a GPS signal input; the results of this proposal have shown the capability of CTS to reach perpetual time synchronization levels of less than 100 ns. This technique is applicable anywhere on Earth where muons can arrive, including regions underground and underwater.

## Results

### Principle of cosmic time synchronization

**Figure 1:**
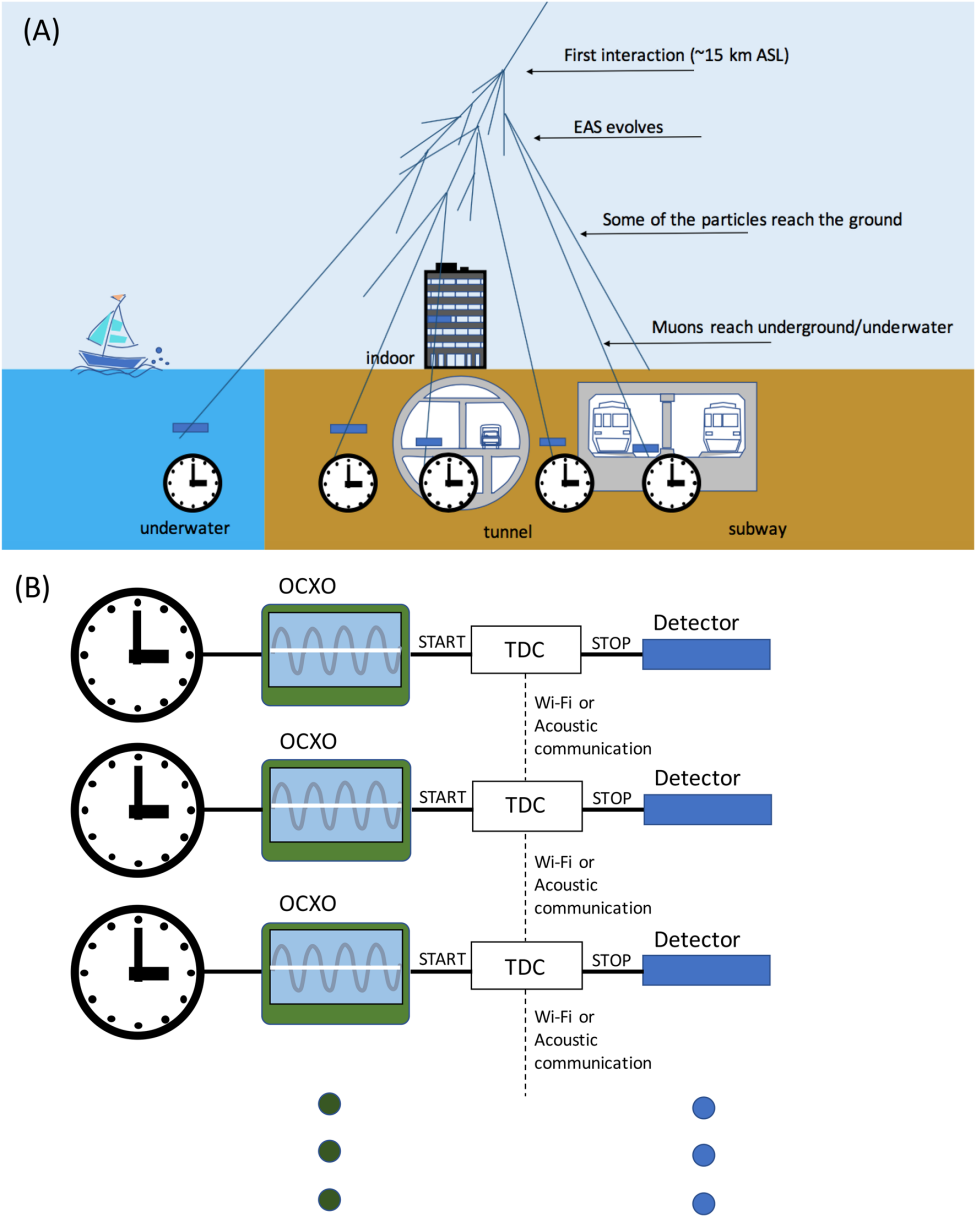
Principle of CTS. The overall CTS system configuration is shown with the EAS developments (**A**). The blue rectangular boxes indicate the CTS modules. The configuration of CTS modules is shown in (**B**). HKMT drew this image and holds the copyright.

Figure 1 shows the principle of CTS. The CTS modules are spatially dispersed within targeted areas for time synchronization; these CTS modules can be placed on the ground surface, underground or underwater. The CTS module consists of a muon detector, a time to digital converter (TDC), and the OCXO. EAS muons arrive at the ground almost simultaneously with a certain measurable spatial extent. These muons are relativistic and therefore they have sufficient energy and lifespan to penetrate dense materials: depending on energy levels, a sizable proportion will travel through the Earth and bodies of water to reach underground and underwater regions. The arrival depth of a muon depends on its kinetic energy; for example, 10-GeV muons can reach the seafloor as far as down as 43 m. The detectors output signals when muons pass through them. Detectors consist of plastic scintillators and photodetectors: inexpensive components with an efficient time response. The output signals from the detectors are given to the TDC as the stop signals. On the other hand, the signals from the OCXO are given to the TDC as the start signals so that the time difference between the OCXO and the muon's arrival time can be measured. If more than two CTS modules are triggered (for the purpose of this example, labeled as CTS Module 1 and CTS Module 2) within the given time window (*T*) (hereafter this coincidence is defined as a local coincidence (LC)), the time stamp recorded in the TDC of CTS Module 1 would be transferred to CTS Module 2. When CTS Module 2 receives the time stamp from CTS Module 1, this time stamp is compared with the local time of CTS Module 2 and can be then used for correcting the local time of CTS Module 2. The coincidence window between CTS Module 1 and CTS Module 2 doesn’t have to be narrow as described later. More specifically, although the CTS Module 1 and CTS Module 2 receive the EAS muons at an absolute time *t*_0_, this time could be registered as *t*_0_ + δ*t*_1_ + Δ*t*_1_ at CTS Module 1 and as *t*_0_ + δ*t*_2_ + Δ*t*_2_ at CTS Module 2 due to uncertainties (δ*t*) coming from the EAS time structure and the OCXO’s intrinsic drift (Δ*t*). By transferring the time information *t*_0_ + Δ*t*_1_ from CTS Module 1 to CTS Module 2, CTS Module 2 can calculate (*t*_0_ + Δ*t*_2_ + δ*t*_2_) − (*t*_0_ + Δ*t*_1_ + δ*t*_1_) = (Δ*t*_2_ − Δ*t*_1_) + (δ*t*_2_ − δ*t*_1_) for the correction of its clock. For this calculation, neither *t*_0_, Δ*t*_2_ nor Δ*t*_1_ are necessary to define. As is described later, (δ*t*_2_ − δ*t*_1_) is typically less than 50 ns and is also much smaller than the typical OCXO’s drift rate (up to a few µs/hr). Moreover, the data transfer time (~ 0.1 ms) required for identifying coincidence events is likely to be attainable, and is negligible in comparison to a shower frequency of every 10 min that will be described in detail in the next subsection.

The time synchronization input of the CTS's synchronizing frequency depends on the EAS frequency since the clock is corrected only when the EAS muon arrives at the detectors, and the synchronization accuracy depends on the EAS time structure (muon's arrival time distribution). Additionally, the synchronization capability within the intervals between EAS arrivals depends on the OCXO drift level. Although each CTS module should be part of a conventional Wi-Fi or the acoustic communication network, the CTS's synchronization accuracy is independent from their intrinsic jitter and the latency.

If each CTS module has a detection area of 1 × 1 m^2^, the single muon count rate at each detector will have an expected rate of 10^2^ Hz at sea level. Therefore, the accidental twofold and threefold LC rates will be respectively 10^−2^ Hz and 10^−6^ Hz with a coincidence window of 100 microseconds. This required *T* is much wider (100 microseconds) than the legacy WLAN^3^ capability. With a threefold LC request, statistically the CTS module is likely to receive the wrong time stamp every 10 days. In order to reduce this time synchronization failure rate, we need to either narrow the time window or request a simultaneous hit larger than a threefold LC. Whether the narrower time window can be used solely depends on the synchronization accuracy of the Wi-Fi or the underwater acoustic technique used. For example, for a land-based wireless communication, synchronization accuracy of 10 microseconds is relatively easy to achieve^3^, but for the underwater acoustic technique, achieving this accuracy is more challenging due to unexpected signal propagation properties in water (temperature, salinity, etc.)^41^. Requesting an LC larger than threefold may be a simpler solution to reduce this failure rate. For example, if we request fourfold LC instead threefold LC, it would take approximately 3 years before the module would receive its first wrong time stamp after operating without a GPS signal. As a consequence, the failure rate due to the accidental LC is negligible when using a dense CTS network (more than 4 CTS nodes within the unit area).

### Muon lateral distribution and time structure

The average muon Lateral Distribution Function (LDF) can be described by Greisen’s function^42^:

1 $$\rho_{\mu } \left( r \right) = \rho_{\mu } \left( {r_{0} } \right)\left( {\frac{r}{{r_{0} }}} \right)^{ - 3/4} \left( {\frac{320 + r}{{320 + r_{0} }}} \right)^{ - \gamma }$$

as a function of lateral distance, where the lateral distance is defined as the distance from the shower axis. Here, the first exponent of *r* was fixed to -3/4^43^. Several experiments have attempted to fit the parameters gamma and *ρ*_*µ*_(*r*_0_)^44–46^. In this work, the results obtained at IceTop^43^ were employed. IceTop is a detector array consisting of 81 stations forming a grid with a separation of 125 m, covering an area of ~ 1 km^2^. Each station consists of 2 ice-tank-based Cherenkov detectors separated by 10 m^47^. In this work, Greisen’s function based on IceTop *ρ*_*µ*_(600)^43^ was used as an initial input.

**Figure 2:**
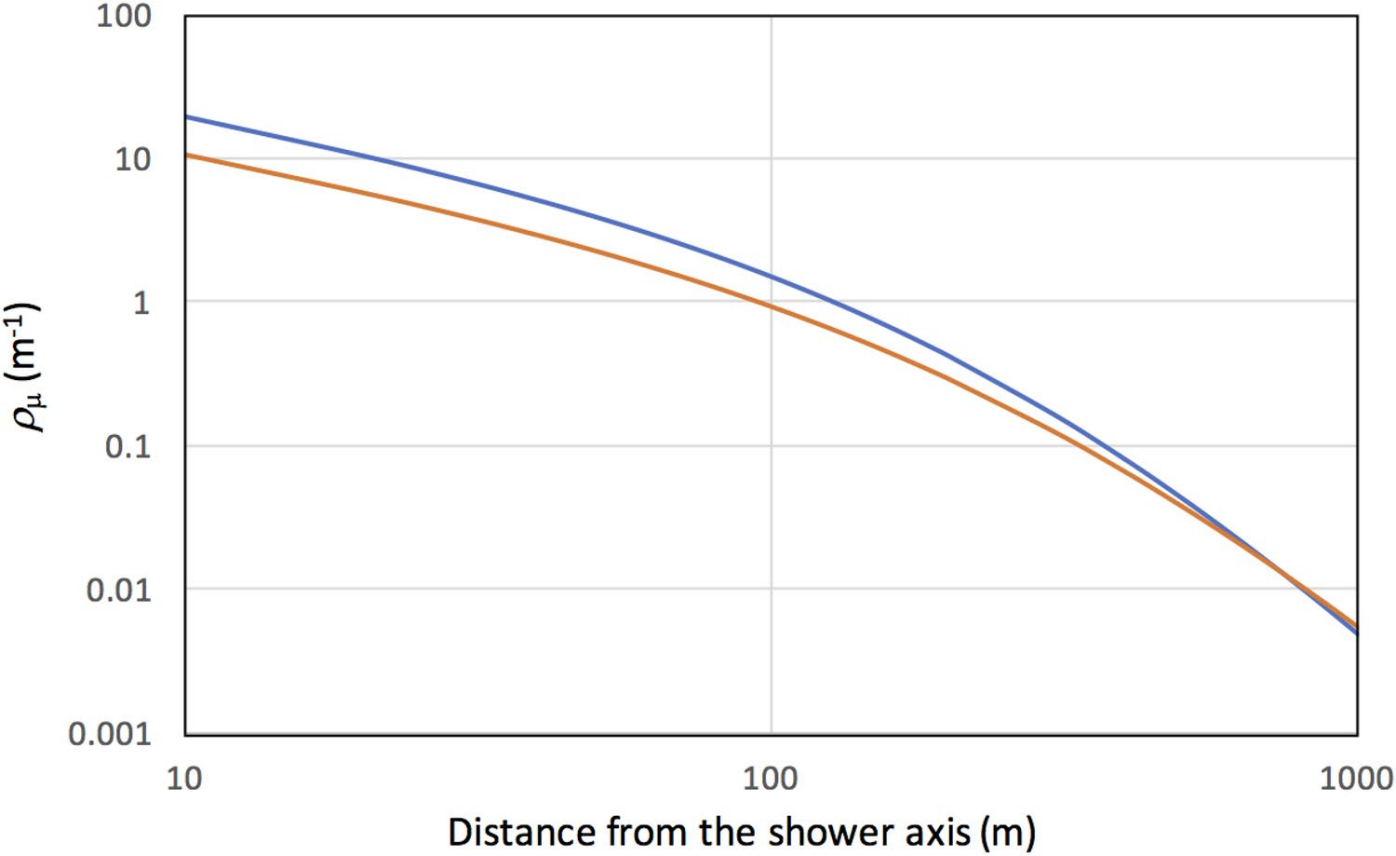
Greisen function curves fitted to the IceTop data at zenith angles of less than 6° (blue solid line) and those at zenith angles between 28° and 31° (orange solid line)^43^.

In Fig. 2, Greisen’s function curves for the showers initiated by the primary cosmic rays with energies at 10 PeV are shown. These curves were a result of fitting the IceTop data at zenith angles of less than 6° and also with data at zenith angles between 28° and 31°^43^. As can be seen in this figure, *ρ*_*µ*_ exceeds 1 muon/m^2^ within the area of 140 m from the shower axis for the vertical shower initiated by primaries with energies greater than 10 PeV. However, not all shower axes are oriented vertically. As can be seen in Fig. 2, smaller *ρ*_µ_ would be observed in the slanted showers. For example, the area at which *ρ*_*µ*_ exceeds 1 muon/m^2^ is reduced from 140 to 100 m from the shower axis for the shower arriving at zenith angles between 28° and 31°.

In order to evaluate the attainable synchronization accuracy of CTS, the primary spectrum^48^ must be considered. The integrated flux of the primaries with energies greater than 10 PeV is 10^2^ km^−2^ h^−1^ sr^−1^ but its flux is reduced to the power of 2 as energy increases, and becomes 1 km^−2^ h^−1^ sr^−1^ for the primaries with energies greater than 100 PeV. Within a region with a radius of 140 m (6 × 10^4^ m^2^), for example, it is expected that primaries with energies greater than 10 PeV arrive every 10 min; hence the clock can be synchronized every 10 min if we can use this 10-PeV EAS for synchronization. It is impractical to use EAS initiated by the primary energies below 1 PeV since this would make the *ρ*_*µ*_ too low. Likewise, it is also impractical to use the primary energy region above 100 PeV since the event frequency would be too low.

**Figure 3:**
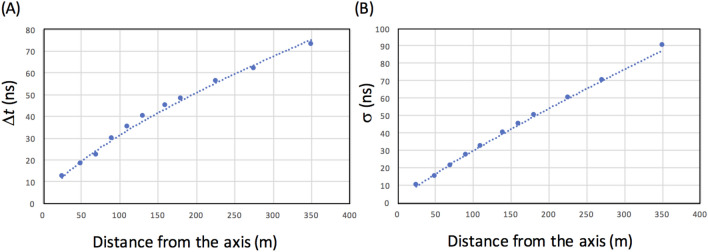
Time structure of the extended air shower initiated by the primaries with energies greater than 10 PeV. The EAS average arrival time (Δ*t*) (**A**) and the disk thickness (*σ*) (**B**) are shown as a function of the distance from the shower axis. Filled circles indicate the experimental results as obtained with the KASCADE experiment^33^.

The KASCADE collaboration measured that the average EAS arrival time (Δ*t*) and the disk thickness (*σ*) (standard deviation of the particle arrival time distribution) depend on the distance from the shower axis (Fig. 3)^33^. These lateral distance dependencies were fitted by the following power function (dotted line in Fig. 3), and used for the current discussion.

2 $$\Delta t = { 1}.{399}r^{{0.{6743}}}$$

3 $$\sigma = \, 0.6058r^{0.8455}$$

The maximum deviation of the fitted value from the observed value was 2 ns and this deviation was neglected in the current modeling.

### OCXO evaluation

**Figure 4:**
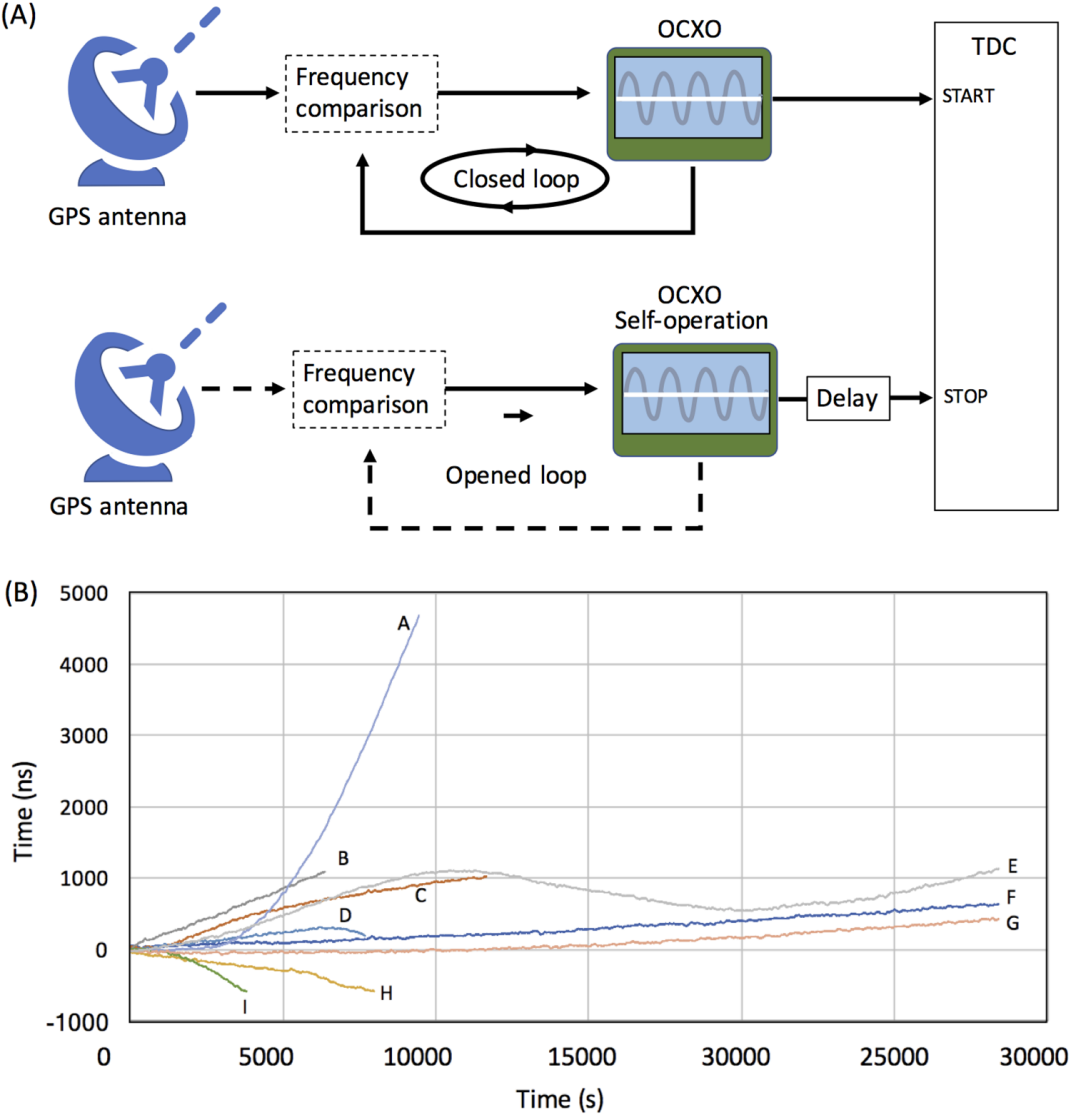
Results of the current OCXO evaluation. The block diagram of the current experimental setup is shown in (**A**). The dashed lines indicate the disconnected flows. The OCXO drift was measured as a function of the time after disconnection from the GPS antenna for various durations of the GPS reception before disconnection (**B**): A, 0.5 h, B, 48 h, C, 72 h, D, 16 h, E, 10 min, F, 0 h (no antenna connections), G, 8.5 h, H, 16 h, I, 1 h. HKMT drew this image and holds the copyright.

Oven Controlled Crystal Oscillators (OCXO) such as the Single-Oven Controlled Oscillator (SOCO) or the Double-Oven Controlled Oscillator (DOCO) were developed for the improvement of long-term timing stability^27^. OCXO has been used for providing backup timing signals when a GPS signal is lost. In this work, the speed and behavior of the OCXO drift caused by the accumulating errors from inaccuracy in initiating PPS signals was evaluated for application to the current CTS modeling. The OCXO used in this work existed inside the GPS grandmaster clock (Trimble Thunderbolt PTP GM200). Figure 4A shows the experimental setup (More details will be provided in the Method section). By feeding both of the signals from the OCXO connected to the GPS antenna (GPS-OCXO) and signals from the holdover OCXO to the TDC (HLD-OCXO), the drift level of HLD-OCXO timing was measured as a function of time relative to the GPS-OCXO timing as a reference. HLD-OCXO was initially synchronized with GPS-OCXO by connecting a GPS antenna, and then was disconnected before these measurements. A delay circuit was inserted between the OCXO and TDC so that both positive and negative drifts could be measured. Figure 4B shows the obtained timing profile of HLD-OCXO for various durations of the GPS reception before disconnection. In these 9 runs, the drift tended to occur in the forward direction (faster than GPS-OCXO), but it also occurs in the backward directions (slower than GPS-OCXO). If the OCXO operation time is less than 1 h, the drift is roughly linear as a function of time, but for longer operation, the behavior is unpredictable (for example, Run ID E in Fig. 4B).

## Discussion

### CTS modeling

Here, synchronization stability and accuracy will be discussed based on a simple modeling work. This modeling work was dedicated to estimating the minimum achievable performance of CTS, and is not intended to address precise EAS structures. For this modeling work, the following conditions were used.

(A) Estimating the intervals of the EAS that can be used for CTS. For this, only the showers initiated by the primaries with energies above 10 PeV arriving within the vertical angular region of 1 sr were considered. The frequency of such events is 10^2^ km^2^ hr^−1^ sr^−1^. For the purpose of estimating the minimum frequency of time synchronization, this condition would be sufficient.
(B) Estimating *ρ*_µ_. For this objective, Greisen function curves based on the 10-PeV IceTop results at zenith angles between 28° and 31° were employed. By choosing these angles, *ρ*_µ_ will be slightly underestimated, however, this condition would be sufficient for estimating the minimum available *ρ*_µ_. Using a combination of the primary flux integrated over the range above 10 PeV and the 10-PeV EAS muon lateral distribution would be sufficient for the current purpose since higher muon multiplicity is expected for higher primary energies; hence this combination provides data on the minimum amount of available *ρ*_µ_.
(C) Assumptions for CTS coverage. The following three cases were assumed: (Case A) 5 × 10^−5^, (Case B) 1 × 10^−4^, and (Case C) 2 × 10^−4^.
(D) Average arrival time and its fluctuation. These functions were respectively generated based on Eqs. (2) and (3) and the distance between the CTS module and the shower axis was also randomly generated within the range between 0 and 100 m.
(E) OCXO Reset. Every time an EAS event was generated, the OCXO was reset, and the time profiles were generated for comparison between CTS modules.

**Figure 5:**
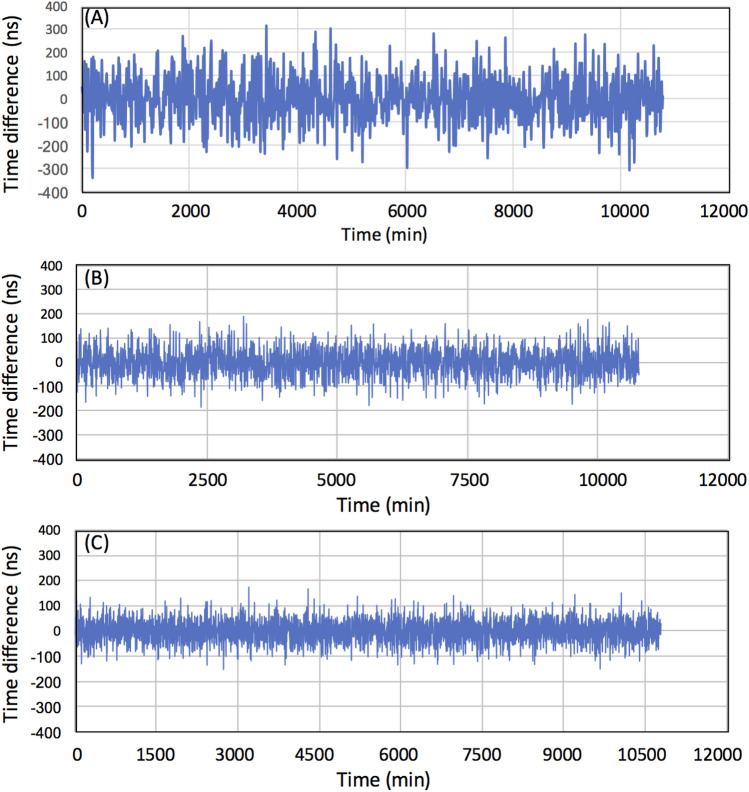
Time profiles after 1 week of CTS operation. The results with hypothetical detector arrays of areal coverages 5 × 10^−5^ (**A**), 1 × 10^−4^ (**B**), and 2 × 10^−4^ (**C**) are shown.

Figure 5 shows the time profiles after 1 week of operation to find the difference between CTS Module 1 and CTS Module 2, assuming different areal fractions of the module occupation. The standard deviations for Cases A, B, and C were respectively found to be 98 ns, 56 ns, and 42 ns with the maximum deviation of 314 ns, 189 ns, and 174 ns. It is anticipated that this accuracy will be maintained during for much longer operation periods.

### Prospects

In principle, this recurrent time synchronization processes can be repeated forever without GPS signals, as long as primary cosmic rays and the Earth's atmosphere exist. This EAS-based non-GPS synchronization input could be implemented worldwide in almost every location including underground and underwater. Although we need a relatively high density array of CTS modules, it is anticipated that CTS will be adopted as a new type of time synchronization tool in polar, underwater and underground environments where GPS signals are not available or only partially available.

Downgrading of time synchronization accuracy is generally due to (A) the OCXO’s frequency drift and (B) the EAS’s time structure. For (A), the synchronizing accuracy of the proposed method can be improved with more stable clock technology. As can be seen in Fig. 5, the synchronization accuracy depends on the CTS areal coverage; hence the OCXO correction frequency. Better CTS areal coverage is a tradeoff with the stability of the clock and vice versa. One of the simplest solutions to improve the clock stability is usage of multiple OCXOs^49^. The random behavior of the frequency drift will be partially canceled out by taking average of the multiple OCXOs signal outputs; hence the clock stability will be improved. Since the costs of the OCXOs are in the order of 100 dollars, this could be a reasonable option. For (B), as can be seen in Fig. 3, variations in arrival times of muons tend to be suppressed as they get closer to the shower axis; hence the time synchronization accuracy will be improved further if we use only the muons near the axis, but a synchronization accuracy of 10 ns is the practical limit of this technique. Moreover, since the area used for synchronization is reduced, CTS modules must be more densely located.

One single CTS action only works within the area EASs cover on ground level. If the CTS technique is combined with more stable clocks such as a Cs oscillator that enables a 100-ns maximum time interval error (MTIE) over 14 days in Holdover mode (Class-A)^50^, less frequent but larger EASs initiated by higher primaries can be utilized for time synchronization. For example, since the EASs initiated by a few-hundred-PeV primaries contain charged particles with number densities higher than 1-m^−2^ at ground level even at locations 500 m from the shower axis, the CTS modules could be located at a km-order intervals. However, the purpose of the current work is to propose a globally-applicable, inexpensive but practical time synchronizing system. A Cs oscillator is still expensive (a few hundred thousand dollars) and sensitive to the ambient temperature variations. Moreover, as shown in Fig. 3, since the arrival time and thickness of the EAS particles depend on the distance from the shower axes, longer intervals between CTS modules would degrade the synchronization accuracy. On Earth, 14 million EASs are generated every second by the primaries with energies greater than 10 PeV. Therefore, an action chain of a number of single CTS actions enable global time synchronization. The results as obtained in the previous CTS modeling works indicate that for example, one in NY, and one in LA can be synchronized by adding an appropriate number of CTS modules between them.

In indoor or underground environments, the world-wide network composed of billions of consumer smartphones might play a large role in the near future. It has already been verified that CMOS-based cameras attached to smartphones had sufficient capability to detect muons^51^. Although each device has a limited detection efficiency (ranging from 70 to 90%)^52^, together as a network the smartphones have a potential to be CTS modules. The detection area available on smartphone is very small (0.2 cm^2^)^51^, however one way to solve this problem could be to take advantage of the large number of people that are often present inside skyscrapers. For example, a moderately large skyscraper (with a base area of 10^4^ m^2^ and a height of 200 m) can usually accommodate 50,000 people^53^. Therefore, if we assume each individual inside the aforementioned example skyscraper has one smartphone, the CTS coverage would be 10^−4^. Although skyscrapers usually have a GPS antenna at the top, CTS can be used as a backup system when GPS signals are not available for some reason such as multipath reception, GPS jamming and spoofing, and system failures, etc. There are limitations in the multiple coincidence time window due to the poor time resolution determined by the cellphone camera exposure duration (milliseconds to seconds)^54^. However, the detection area of the smartphone camera is sufficiently small (0.2 cm^2^), and thus the multiple accidental coincidence is unlikely to occur since the single muon counting rate would be extremely low (1 muon count every five minutes). In this scenario, small numbers of CTS modules should be located somewhere inside the building for required time synchronization inside the building. Multiple coincidence rates of smartphones would be used for triggering CTS and the generated timestamp would be transferred to another CTS module to synchronize.

Another possibility to both benefit and share the CTS time synchronization capability would be time synchronization operation in a dense underwater sensor network (USN) (more than a few hundred nodes per square meter). For example, this kind of dense USN could be associated with the Robotic Vessels as-a-Service (RoboVaaS) project, which is a new scheme aiming at revolutionizing near-shore operations in coastal waters by integrating and networking a smaller Unmanned Surface Vehicle (USV) and an Unmanned Underwater Vehicle (UUV) efficiently in order to offer new services for shipping^55^. In order to safely operate RoboVaaS, the environmental data are collected by a dense USN, which will inspect the impact to RoboVaaS, as well as to monitor the coastal conditions^56^. Conventionally, the deployment of a dense USN has been unrealistic since the costs for acoustic communications have been high (~ 10 k USD) and thus, has been typically limited to use in critical areas and military applications, where these costs can more easily be justified. Recently, this situation has been greatly improved. It was reported that the new high frequency smartPORT acoustic modem (AHOI) (600 USD)^57^ is expected to enable dense USN (> 500 nodes/km^2^) to be employed in civil applications, as its overall cost is one order of magnitude cheaper than the conventional one. If we assume a scenario in which every node of such a dense USN would be equipped with CTS, the required CTS detection area would be < 2000 cm^2^. Then the size of each CTS module could be approximately ISO 216 B3, and the cost would be approximately 600 USD (scintillator, SiPM and WLS fiber) for each module. Moreover, by combining the CTS modules and the newly developed muometric positioning system (muPS)^40^, wireless passive positioning could also be possible.

Also, it is anticipated that CTS would function well in underground environments such as mine galleries, subway station complexes, and underground parking lots. Positioning systems in these environments would require accurate measurements of the time of arrival of a transmitted signal and therefore precise time synchronization would be another requirement. Wi-Fi signals transmitted in underground tunnels for clock synchronization would be hampered with severe multipath effects caused by reflection and refraction. Upgrading these systems from Wi-Fi to CTS would solve this problem.

In conclusion, CTS has been proposed as a new technique for GPS-free, temperature-independent accurate time synchronization and the requirements for practical application were also discussed. The EASs initiated by the primaries with energies above 10 PeV could be utilized for practical CTS applications. CTS aerial coverage of 10^−4^ would be required. This aerial coverage would be achievable with strategies such as taking advantage of a dense smartphone network and/or sharing an underwater sensor network associated with the RoboVaaS project; the attainable synchronization accuracy would be under 100 ns. CTS has strong potential to be the next high-precision time synchronization technology standard that can support successful implementation of emerging technologies, such as 5G technology, in the near future.

## Method

### Experimental setup

In the current modeling work, two CTS modules were developed. For the current modeling purpose, deployment of a large-scale detector array was not intended and thus, muon detectors were not equipped to the CTS modules. Therefore, the current CTS modules consisted of a GPS grandmaster clock (Trimble Thunderbolt PTP GM200), a TDC (Sciosence TDC-GPX), a complex programmable logic device (CPLD), and Raspberry Pi. Two independent PPS (pulse per second) signals were generated by the two GPS grandmaster clocks, and the OCXO frequency difference between these two clocks were measured by the TDC. The PPS signals from GPS-OCXO and HLD-OCXO were converted to the NIM level and transferred to the TDC as a start and stop signals, respectively. Since both positive and negative drifts were expected, a delay circuit (600 ns) was inserted between HLD-OCXO and the TDC. The signals from the TDC were transferred to a CPLD, and subsequently transferred to Raspberry Pi for communication to the local PC via Ethernet. The measurable time range of the TDC was 10 microseconds with a resolution of 27 ps, and the minimum receivable pulse width was 10 ns.

## References

[1] Mahmood A (2019). Time synchronization in 5G wireless edge: requirements and solutions for critical-MTC. IEEE Commun. Mag..

[2] Shi *et al.* Evaluating the performance of over-the-air time synchronization for 5G and TSN integration (2021). Retrieved from https://arxiv.org/abs/2104.13873.

[3] Hiyama, M. *et al.* Implementation of precision clock synchronization protocol to IEEE802.11 (2009). Retrieved from https://ipsj.ixsq.nii.ac.jp/ej/?action=repository_action_common_download&item_id=61816&item_no=1&attribute_id=1&file_no=1.

[4] Milevsky A (2008). Development and test of IEEE 1588 precision timing protocol for ocean observatory networks. Oceans.

[5] IEEE 802.1 Time-Sensitive Networking (TSN) Task Group (2021). Retrieved from http://www.ieee802.org/1/ pages/tsn.html.

[6] Rost M (2012). Time transfer through optical fibers over a distance of 73 km with an uncertainty below 100 ps. Metrologia.

[7] Sliwczynski L (2013). Dissemination of time and RF frequency via a stabilized fibre optic link over a distance of 420 km. Metrologia.

[8] Gersl J (2015). Relativistic corrections for time and frequency transfer in optical fibers. Metrologia.

[9] Krehlik P (2016). ELSTAB- fiber optic time and frequency distribution technology: a general characterization and fundamental limits. IEEE Trans. Ultrason. Ferroelect. Freq. Control.

[10] Dierikx E (2016). White rabbit precision time protocol on long-distance fiber links. IEEE Trans. Ultrason. Ferroelectr. Freq. Control.

[11] Cavalcanti D (2019). Extending accurate time distribution and timeliness capabilities over the air to enable future wireless industrial automation systems. Proc. IEEE.

[12] Aijaz A (2020). High-performance industrial wireless: achieving reliable and deterministic connectivity over IEEE 802.11 WLANs. IEEE Open J. Ind. Electron. Soc..

[13] Esteban H (2010). Improved GPS-based time link calibration involving ROA and PTB. IEEE Trans. Ultrason. Ferroelect. Freq. Control.

[14] Valat D, Delporte J (2020). Absolute calibration of timing receiver chains at the nanosecond uncertainty level for GNSS time scales monitoring. Metrologia.

[15] Jiang Z (2019). Improving two-way satellite time and frequency transfer with redundant links for UTC generation. Metrologia.

[16] Gundall, M. *et al.* Integration of IEEE 802.1AS-based Time synchronization in IEEE 802.11 as an enabler for novel industrial use cases. arXiv:2101.02434v1 (2021).

[17] Mahmood A (2017). Clock synchronization over IEEE 802.11—a survey of methodologies and protocols. IEEE Trans. Ind. Inform..

[18] Cena G (2015). Implementation and evaluation of the reference broadcast infrastructure synchronization protocol. IEEE Trans. Ind. Inform..

[19] Chang T (2015). Adaptive synchronization in multi-hop TSCH networks. Comput. Netw..

[20] Yang Z (2014). Temperature-assisted clock synchronization and self-calibration for sensor networks. IEEE Trans. Wirel. Commun..

[21] Carli R, Zampieri S (2014). Network clock synchronization based on the second-order linear consensus algorithm. IEEE Trans. Autom. Control.

[22] Masood W (2016). Dynamic stochastic time synchronization for wireless sensor networks. IEEE Trans. Ind. Inform..

[23] Elson J (2002). Fine-grained network time synchronization using reference broadcasts. ACM SIGOPS Oper. Syst. Rev..

[24] Stojanovic M, Webster JG (1999). Underwater acoustic communication. Wiley Encyclopedia of Electrical and Electronics Engineering.

[25] Frank, M. P. *et al.* Design of a wireless sensor network with nanosecond time resolution for mapping of high-energy cosmic ray shower events. In *Proc. SPIE 7706, Wireless Sensing, Localization, and Processing V*, p. 770603 (26 April 2010). 10.1117/12.852334.

[26] Zhan L (2016). Utilization of chip-scale atomic clock for synchrophasor measurements. IEEE Trans. Power Deliv..

[27] Frederic, L. *et al*. A new kind of view for a double oven crystal oscillator, (2007). Retrieved from https://ieeexplore.ieee.org/document/4319182.

[28] Kampert, K. H. & Unger, M. Measurements of the cosmic ray composition with air shower experiments (2012). Retrieved from https://arxiv.org/pdf/1201.0018.pdf.

[29] Bellido JA (2018). Muon content of extensive air showers: comparison of the energy spectra obtained by the Sydney University Giant Air-shower Recorder and by the Pierre Auger Observatory. Phys. Rev. D.

[30] Antoni T (2001). Time structure of the extensive air shower muon component measured by the KASCADE experiment. Astropart. Phys..

[31] Ambrosio M (1999). Time structure of individual extensive air showers. Astropart. Phys..

[32] Takeda M (1999). Small-scale anisotropy of cosmic rays above 10^19^eV observed with the Akeno Giant Air Shower Array. Astrophys. J..

[33] Apel WD (2008). Time structure of the EAS electron and muon components measured by the KASCADE–Grande experiment. Astropart. Phys..

[34] Tanaka HKM (2007). High resolution imaging in the inhomogeneous crust with cosmic-ray muon radiography: the density structure below the volcanic crater foor of Mt. Asama, Japan. Earth Planet. Sci. Lett..

[35] Tanaka HKM, Kusagaya T, Shinohara H (2014). Radiographic visualization of magma dynamics in an erupting volcano. Nat. Commun..

[36] Tanaka HKM, Aichi M, Bozza C (2021). First results of undersea muography with the Tokyo-Bay Seafloor Hyper-Kilometric Submarine Deep Detector. Sci. Rep..

[37] Tompson LF (2020). Muon tomography for railway tunnel imaging. Phys. Rev. Res..

[38] Oláh L (2012). CCC-based muon telescope for examination of natural caves. Geosci. Instrum. Method Data Syst..

[39] Morishima K (2017). Discovery of a big void in Khufu’s Pyramid by observation of cosmic-ray muons. Nature.

[40] Tanaka HKM (2020). Muometric positioning system (μPS) with cosmic muons as a new underwater and underground positioning technique. Sci. Rep..

[41] Pallarés Valls, O. Time synchronization in underwater acoustic sensor networks (2016). Retrieved from http://hdl.handle.net/2117/105563.

[42] Greisen K (1960). Cosmic ray showers. Annu. Rev. Nucl. Part. Sci..

[43] Gonzalez, J. G. Measuring the muon content of air showers with IceTop (2015). Retrieved from https://arxiv.org/abs/1501.03415.

[44] Abu-Zayyad T (2000). Evidence for changing of cosmic ray composition between 10^17^ and 10^18^ eV from multicomponent measurements. Phys. Rev. Lett..

[45] Nagano M, Hara T, Hatano Y (1984). Energy spectrum of primary cosmic rays between 10^14.5^ and 10^18^ eV. J. Phys. G Nucl. Part. Phys..

[46] Hayashida N (1995). Muons (>or=1 GeV) in large extensive air showers of energies between 10^16.5^ eV and 10^19.5^ eV observed at Akeno. J. Phys. G Nucl. Part. Phys..

[47] Abbasi R (2013). IceTop: the surface component of IceCube. Nucl. Instrum. Methods A.

[48] Zyla PA (2020). The review of particle physics. Prog. Theor. Exp. Phys..

[49] Tanaka, H. K. M. Wireless muometric navigation system. Preprint at https://www.researchsquare.com/article/rs-1348393/v1 (2022). 10.1038/s41598-022-13280-4 PMC9203741 35710813

[50] Microchip. TimeProvider 4100 Series Release 2.3. (2021). Retrieved from https://ww1.microchip.com/downloads/en/DeviceDoc/00004146.pdf.

[51] Homola, P. *et al.* Cosmic ray extremely distributed observatory. (2020), Retrieved from https://arxiv.org/abs/2010.08351.

[52] Swaney, J. *et al.* Measurement of smartphone sensor efficiency to cosmic ray muons. (2021). Retrieved from https://arxiv.org/abs/2107.06332.

[53] Taguchi A (1997). Urban traffic distribution and elevator capacity in super high buildings. J. Oper. Res. Soc. Jpn..

[54] Vandenbroucke, J. *et al.* Detecting particles with cell phones: the distributed electronic cosmic-ray observatory. (2015). Retrieved from https://arxiv.org/pdf/1510.07665.pdf.

[55] Signori A (2019). Data gathering from a multimodal dense underwater acoustic sensor network deployed in shallow fresh water scenarios. J. Sens. Actuator Netw..

[56] Heidemann J (2012). Underwater sensor networks: applications, advances and challenges. Philos. Trans. R. Soc. A.

[57] Renner, C. & Golkowski, A. J. Acoustic modem for micro AUVs: design and practical evaluation. (2016) Retrieved from https://dl.acm.org/doi/abs/10.1145/2999504.3001076.

